# Classification system for primary care provider eConsults about medications for older adults with frailty

**DOI:** 10.1186/s12875-024-02340-5

**Published:** 2024-04-02

**Authors:** T Schneider, B Farrell, S Karunananthan, A Afkham, E Keely, C Liddy, L. M. McCarthy

**Affiliations:** 1 Leslie Dan Faculty of Pharmacy, University of Toronto, Toronto, Canada; 2 Department of Health Research Methods, Evidence, and Impact, McMaster University, Hamilton, Canada; 3 Bruyère Research Institute, Ottawa, Canada; 4 Department of Family Medicine, University of Ottawa, Ottawa, Canada; 5 School of Pharmacy, University of Waterloo, Waterloo, Canada; 6 Interdisciplinary School of Health Sciences, University of Ottawa, Ottawa, Canada; 7 Ontario Health East, Ottawa, Canada; 8 Department of Medicine, University of Ottawa, Ottawa, Canada; 9 Division of Endocrinology and Metabolism, The Ottawa Hospital, Ottawa, Canada; 10 Ontario eConsult Centre of Excellence, The Ottawa Hospital, Ottawa, Canada; 11 C.T. Lamont Primary Health Care Research Centre, Bruyère Research Institute, Ottawa, Canada; 12 Temerty Faculty of Medicine, University of Toronto, Toronto, Canada; 13 Institute for Better Health, Trillium Health Partners, Mississauga, Canada

**Keywords:** Frailty, Pharmaceutical preparations, Primary health care, Remote consultation, Classification, Potentially inappropriate medication list

## Abstract

**Background:**

Providing primary care for people with frailty can be challenging due to an increased risk of adverse outcomes and use of potentially inappropriate medications which may exacerbate characteristics of frailty. eConsult is a service where primary care providers can receive timely specialist advice for their patients through a secure web-based application. We aimed to develop a classification system to characterize medication-focused eConsult questions for older adults with frailty and assess its usability.

**Methods:**

A classification system was developed and refined over three cycles of improvement through a cross-sectional study of 35 cases categorized as medication-focused from cases submitted in 2019 for patients aged 65 or older with frailty through the Champlain BASE eConsult service (Ontario, Canada). The final classification system was then applied to each case.

**Results:**

The classification system contains 5 sections: (1) case descriptives; (2) intent and type of question; (3) medication recommendations and additional information in the response; (4) medication classification; and (5) potentially inappropriate medications. Among the 35 medication-focused cases, the most common specialties consulted were endocrinology (9 cases, 26%) and cardiology (5 cases, 14%). Medication histories were available for 29 cases (83%). Many patients were prescribed potentially inappropriate medications based on explicit tools (AGS Beers Criteria®, STOPPFall, Anticholinergic Cognitive Burden Scale, ThinkCascades) yet few consults inquired about these medications.

**Conclusion:**

A classification system to describe medication-related eConsult cases for patients experiencing frailty was developed and applied to 35 eConsult cases. It can be applied to more cases to identify professional development opportunities and enhancements for eConsult services.

**Supplementary Information:**

The online version contains supplementary material available at 10.1186/s12875-024-02340-5.

## Introduction

Primary care providers (PCPs) are increasingly expected to care for people with frailty who often have multiple comorbidities, take many medications, and are at higher risk of adverse outcomes and clinical complexity [1, 2]. This can make prescribing difficult. Characteristics of frailty can be exacerbated when patients take potentially inappropriate medications (PIMs) given their associated adverse effects, which can further increase cognitive decline, incontinence, and falls [2]. PCPs are often the leaders for developing multidisciplinary teams who care for older adults [3]. As such, they may benefit from specialist consultation services to assist with managing the complexity of caring for people with frailty [3].

Electronic consultation (eConsult) is a secure, asynchronous web-based tool that allows PCPs such as family physicians and nurse practitioners to access physicians and other health specialists (e.g., pharmacists, chiropodists) for advice [4–6]. The goal of eConsult is to provide timely advice to PCPs and potentially avoid the need for an in-person specialist consultation [4, 5]. After completion of the case, PCPs answer survey questions to provide insight into the value and outcome of the eConsult service [7]. The Champlain BASE® eConsult service has been available in the region of Ottawa, Ontario since 2010 and offers over 150 specialties, including GeriMedRisk: three interdisciplinary services composed of physicians, nurses, and pharmacists for geriatric consults pertaining to medicine, psychiatry, and clinical pharmacology [4, 8, 9]. A recent analysis of eConsult cases for patients with frailty found 41% of questions pertained to drug treatments [10].

There is a lack of information regarding the medication-related topics about which PCPs turn to specialists for help for people living with frailty. Given the complexity and heterogeneity of eConsult data related to medications, there is a unique opportunity to characterize and explore PCPs’ medication-related questions which may be enhanced by a classification system that serves to describe eConsult data. To our knowledge, no such classification system exists. In this study, we developed a classification system to characterize medication-focused eConsults for patients with frailty and assessed its usability in a sample of eConsult cases.

## Methods

The classification system was developed and piloted in medication-related cases for patients with frailty from the Champlain BASE eConsult service. Relevant cases were identified from eConsult cases submitted to the service in 2019 for patients aged 65 or older described by the PCP as ‘frail’ [10]. Cases were included in the current study if they were related to medications.

### Classification system development

**Fig. 1:**
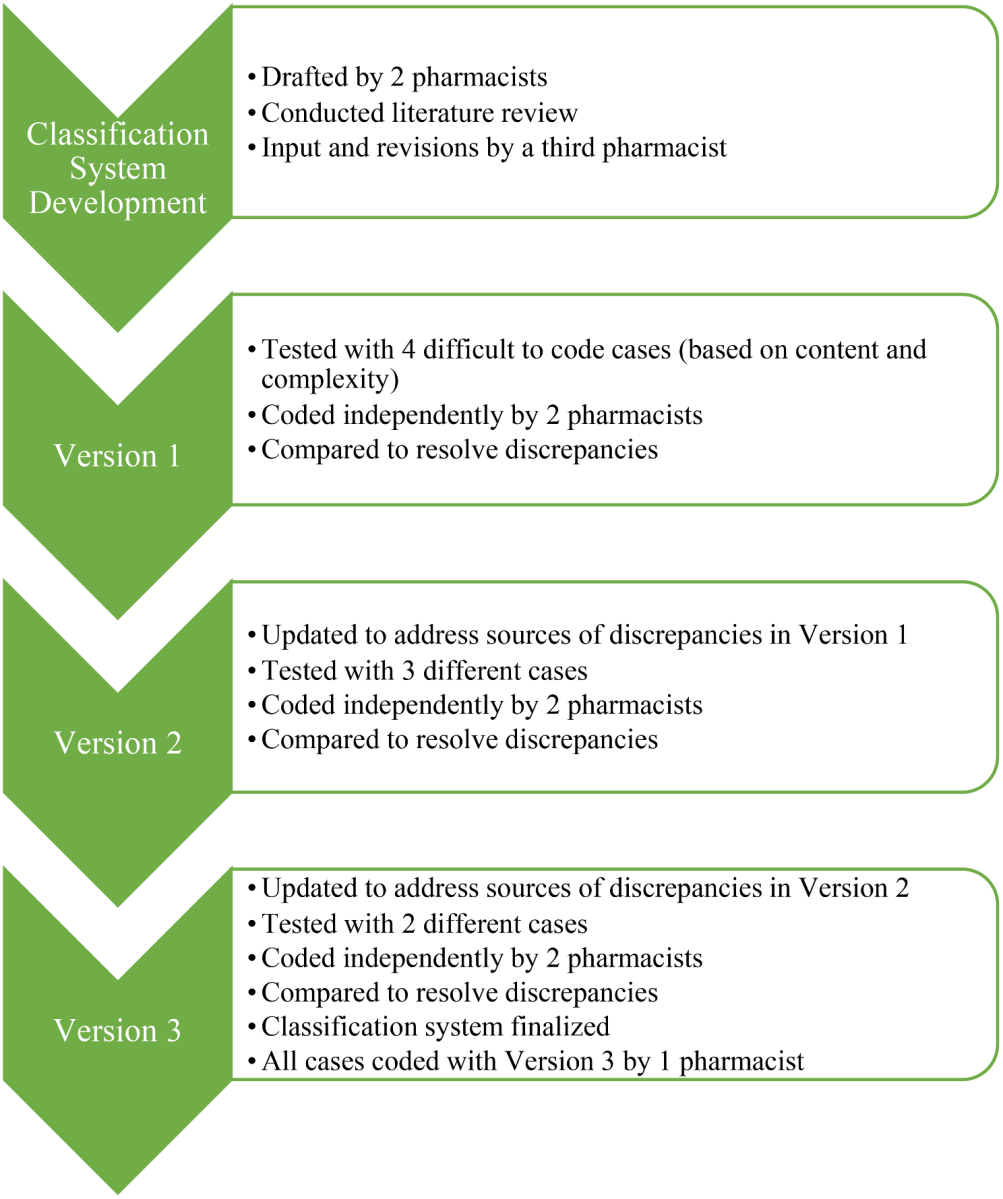
Classification system development process

The development of the classification system was an iterative process that included a literature review, expert consultation, and multiple rounds of testing on a sample of eConsult cases (Fig. 1). The classification system was first drafted by 2 pharmacists (TS, LM) following literature review, then examined by a third pharmacist (BF) who proposed revisions which were discussed for agreement to create the first version of the classification system. Next, 4 cases perceived to be difficult to code based on the content in the case and complexity of questions asked by the PCP were selected from the dataset to be coded independently by 2 pharmacists (TS, LM). The pharmacists met to discuss coding discrepancies and refined the classification system by clarifying descriptions, adding examples, and adjusting categories. The second version of the classification system was applied to 3 new cases and then updated through discussion. The third version was applied to 2 more cases at which point the coders agreed the classification system was finalized. All cases were then coded with the final classification system by 1 pharmacist (TS).

### Data collection and analysis

The classification system was used to collect information about eConsults including case demographics, the questions and responses including format and content, medications involved in the eConsult, and the presence of PIMs in the eConsult and in patient medication histories. Survey answers were also reviewed to better understand the perception of eConsult from PCPs. After applying the final classification system, descriptive statistics for categorical variables (frequencies, percentages, minimums, and maximums) and continuous variables (mean and standard deviation) were calculated.

## Results

### Cases

In 2019, there was a total of 61 eConsult cases about older adults with frailty, where 35 were medication-related.

### Classification system

**Fig. 2:**
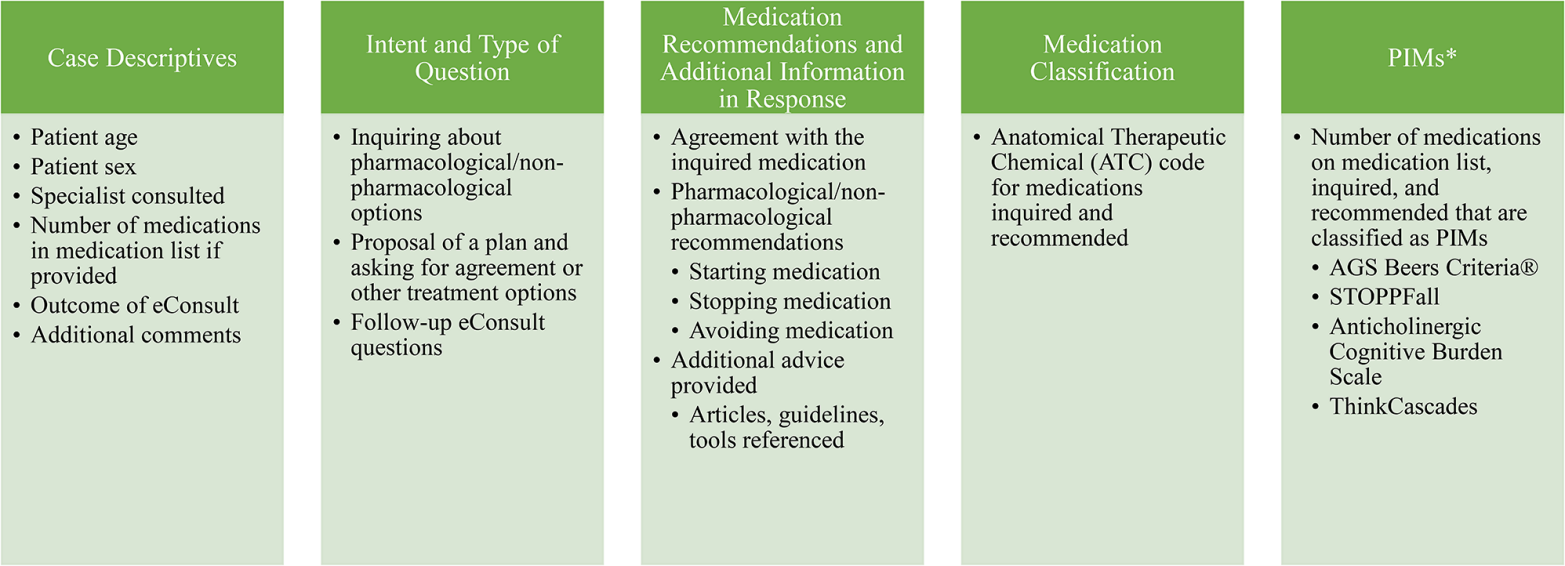
Classification system sections and selected topics. *PIM - potentially inappropriate medication

The resulting classification system contained 5 main sections: (1) case descriptives; (2) intent and type of question; (3) medication recommendations and additional information in the response; (4) medication classification; and (5) PIMs (Fig. 2). Case descriptives extracted from the eConsult cases included patient age, gender, and number of medications on their medication list (if a medication list was provided), as well as type of PCP (physician or nurse practitioner) requesting the eConsult. The intent and type of the question was determined by assessing if the PCP proposed a plan and asked for agreement, proposed a plan and asked for additional options, or did not propose a plan and asked for next steps. The information in the specialist’s response was assessed by whether they recommended a medication to be started, stopped, or avoided, including deprescribing, and if additional information was provided beyond answering the question. Medications were classified with their Anatomical Therapeutic Chemical (ATC) codes [11]. Well-known expert consensus lists of PIMs were consulted to identify PIM use; namely, the American Geriatrics Society (AGS) Beers Criteria® for Potentially Inappropriate Medication Use in Older Adults, the STOPPFall criteria, and the Anticholinergic Cognitive Burden scale, as well as the newer ThinkCascades tool [12–15]. The full classification system is available in Supplemental Table 1, and questions tested but removed from the classification system due to infeasibility are available in Supplemental Table 2.

### Example cases

Summaries of select eConsult cases in this study can be found in Table 1. The first example case involves an eConsult sent to a cardiologist inquiring about antihypertensive therapy for a patient with hypertension, potential heart failure, and other comorbidities. The PCP proposes a plan of switching to a different angiotensin-converting-enzyme (ACE) inhibitor or adding another medication. The specialist responds asking to confirm if the patient has heart failure, but agrees that better control of the patient’s blood pressure is needed. The specialist advised to avoid medications that can increase blood pressure and check that the patient is adherent to their antihypertensive medications. The specialist recommended a calcium channel blocker to be added.

**Table 1:** Example cases

Question	Response	Coding
Case 1: Combination therapy for hypertension		
• 83-year-old female • Chronic kidney disease, hypertension, diabetes, potential heart failure • Furosemide and lisinopril • Blood pressure 170/80 to 195/95 • Recent referral to nephrology • Question to cardiologist: “Would it be a good option to switch to another ACE inhibitor? Combination therapy?”	• Confirm heart failure • Better blood pressure control is needed • Nephrology will likely advise blood pressure management • Avoid medications that can increase blood pressure, including NSAIDs • Check adherence of medications • Calcium channel blocker could be used	• *Case descriptives.* 83-year-old female patient, consulted cardiology, 2 medications provided, PCP accepted recommendation • *Intent and type of question.* PCP inquired about ACE inhibitors, PCP proposed plan and asked for additional options, PCP did not ask follow-up questions • *Medication recommendations and additional information in response.* Specialist recommended a different medication/class could be started than the medication/class inquired, specialist provided additional advice in the form of non-pharms (avoid drugs that increase blood pressure, check adherence) • *Medication classification.* Medication/class inquired: ACE inhibitor ATC code C09AA. Medication/class recommended: calcium channel blocker ATC code C08 • *PIMs.* Patient taking furosemide (appears on STOPPFall), has ACB score of 1
Case 2: Stroke/TIA prophylaxis		
• 88-year-old male • Recurrent peripheral vertigo, 2 previous strokes • After hospital admission unrelated to stroke, was advised to take ASA in addition to clopidogrel upon discharge • Question to stroke/TIA specialist: “Can you clarify whether he should be on clopidogrel alone or + ASA and what degree of benefit of dual therapy in a frail elderly patient like this for TIA/stroke prevention?”	• Advised that dual antiplatelet therapy is not typically used long-term because of the risk of bleeding • Generally used following an acute event, and single antiplatelet therapy is continued • If the patient has not been on ASA, take ASA • If the patient failed on ASA, then take clopidogrel	• *Case descriptives.* 88-year-old male patient, consulted stroke/TIA specialist, 9 medications provided, PCP accepted recommendation • *Intent and type of question.* PCP inquired about clopidogrel and ASA, PCP proposed plan and asked for agreement, PCP did not ask follow-up questions • *Medication recommendations and additional information in response.* Specialist recommended deprescribing of either ASA or clopidogrel, specialist agreed with one of the classes inquired, specialist answered the question and did not provide additional advice. • *Medication classification.* Medication/class inquired: clopidogrel ATC code B01AC04, ASA ATC code B01AC06. Medication/class recommended: clopidogrel ATC code B01AC04, ASA ATC code B01AC06 • *PIMs.* Patient taking rabeprazole (proton pump inhibitor) on AGS Beers Criteria®

ACB – Anticholinergic Cognitive Burden; ACE – angiotensin-converting-enzyme; ATC – Anatomical Therapeutic Chemical; ASA – acetylsalicylic acid; NSAID – non-steroidal anti-inflammatory drug; PCP – primary care provider; PIM – potentially inappropriate medication; TIA – transient ischemic attack

The second example case involves an eConsult sent to a stroke/transient ischemic attack (TIA) specialist about a patient who has had 2 prior strokes and is taking clopidogrel and acetylsalicylic acid (ASA) for stroke prevention. The patient ambulates with a walker and has experienced falls. The PCP proposes two options for a plan: prescribing clopidogrel monotherapy or dual therapy with ASA, and asks for agreement with one of those options. The specialist responds that dual antiplatelet therapy is typically used acutely and not long-term because of the risk of bleeding, where single antiplatelet therapy is continued. The specialist recommended that if the patient has not taken ASA, then they can take it; however, if they failed on ASA, they should take clopidogrel.

### Case analysis

#### Case descriptives

eConsults were submitted primarily by family physicians (31 cases, 89%), with 4 cases (11%) by nurse practitioners (Table 2). The most common specialties consulted were endocrinology (9 cases, 26%) and cardiology (5 cases, 14%); GeriMedRisk was consulted for their psychiatry and clinical pharmacology specialists in 1 case each (3%). All cases were answered by physician specialists and none were submitted to pharmacists. Of the 35 cases, 24 were submitted for female patients (69%) with a mean age of 84 (SD 10) years. Twenty-nine cases (83%) contained a medication history for the patient, who were prescribed a mean 9 (SD 5) medications.

**Table 2:** Descriptive information of included eConsult cases

Description	Number of Cases [n (%)]
Consulting Provider	
Family physicians	31 (89%)
Nurse practitioners	4 (11%)
Specialty Consulted	
Endocrinology	9 (26%)
Cardiology	5 (14%)
Other	21 (60%)
Female patients	24 (69%)
Age (y), mean (SD)	84 (10)
Medication histories provided (n, %)	29 (83%)
Number of medications on histories, mean (SD)	9 (5)

*SD - standard deviation

#### Intent and type of question

Fourteen cases (40%) involved PCPs proposing a plan and asking for agreement, 12 PCPs (34%) proposing a plan and asking for additional options, and 9 PCPs (26%) not proposing a plan and asking for options.

#### Medication recommendations and additional information in response

Specialists provided additional advice or resources in addition to responding to the PCP question in most cases (*n* = 30, 86%). In 1 case (3%), the specialist recommended a best possible medication history (BPMH) to be conducted by 1 of the pharmacists on their team. In 30 cases (86%), starting a new medication was recommended, 14 cases (40%) suggested avoiding a medication, and 6 cases (17%) recommended deprescribing.

#### Medication classification

Denosumab was the most common medication featured in eConsult cases. It was inquired about, and recommended, in 4 and 5 cases (11% and 14%), respectively.

#### PIMs

Of 29 patients for whom medication histories were provided, many were taking medications on the AGS Beers Criteria® (59%), STOPPFall (76%), or with anticholinergic properties (69%). Eight patients were also taking medication combinations that could suggest a potential prescribing cascade (28%). However, few cases inquired about medications on the AGS Beers Criteria® (12%), STOPPFall (23%), with anticholinergic properties (31%), or potential prescribing cascades (0%) (Table 3).

**Table 3:** Cases involving potentially inappropriate medications

Potentially Inappropriate Medication Tool	Number of Cases with a Patient Taking a Medication Included on the Tool (Percentage of Cases*)	Number of Cases Inquiring About a Medication on the Tool (*n* = 26) (Percentage of Cases*)
AGS Beers Criteria® for Potentially Inappropriate Medication Use in Older Adults	17 (59%)	3 (12%)
STOPPFall	22 (76%)	6 (23%)
Anticholinergic Cognitive Burden Scale	20 (69%)	8 (31%)
ThinkCascades	8 (28%)	0 (0%)

*Based on *n* = 29 cases for which a medication list was supplied

### Survey results

According to the PCPs after closing the eConsults, 16 (46%) originally contemplated referral but had now avoided it, 5 (14%) originally contemplated referral and still needed to refer, 12 (34%) were not contemplating referral and did not need to refer, and 2 (6%) provided other comments. When PCPs described the outcome of the eConsult, 14 (40%) stated they confirmed a plan they already had in mind, 20 (57%) received advice about a new or other plan, and 1 (3%) answered none of the above.

## Discussion

We developed this classification system to describe medication-related eConsult cases to leverage this novel information source for understanding PCP knowledge gaps about prescribing for older adults with frailty. Using a sample of 35 eConsult cases related to medications for patients with frailty, we were able to assess the feasibility of the classification system and its usefulness in identifying question and response patterns related to medications in eConsult.

To our knowledge, this is the first classification system to describe medication-related cases for older adults with frailty. This classification system was adapted from prior eConsult studies to describe this population, including a primary care eConsult service involving pharmacists which classified the topics of questions and recommendations, medication classes, medication related problems, and adverse drug events [16]. Specific concepts including starting or stopping a medication and polypharmacy were modified and implemented in this classification system [16]. Additionally, the pharmacist eConsult service classified medications with the American Hospital Formulary Service Pharmacologic-Therapeutic Classification System, inspiring our team to also use a medication classification system. We selected ATC codes due to it being an international standard [16, 17].

Three main patterns were identified by applying the classification system to eConsult cases, which speaks to the usefulness of the classification system even when applied to a preliminary data set. First, the majority of PCPs proposed a plan at the time of consultation, demonstrating that most were seeking reassurance or other potential options. This finding was consistent when assessed with the classification system and compared to PCP survey responses (e.g., both showed 14 PCPs asking and receiving agreement, and 21 PCPs for the classification system inquired about additional options, compared to 20 receiving them in the survey). Second, very few consults were directed to geriatric services and none to pharmacists despite these patients experiencing frailty, polypharmacy, having high rates of PIM use, and PCPs asking medication-focused questions. Outreach could be undertaken to emphasize the potential value of geriatrics and pharmacy services for people living with frailty, and the importance of BPMHs and medication reviews [9, 18, 19]. Third, many patients were experiencing polypharmacy and taking PIMs, but many consults did not focus on deprescribing or PIMs. This is an example of a potential learning opportunity identified for PCPs, as frailty is a risk factor for polypharmacy and PIMs can exacerbate frailty syndromes including cognitive decline and falls [2, 20].

Few consults inquired about or recommended deprescribing, which is surprising considering the focus of the consults on medication-related concerns about older adults, many of whom can be considered complex. Although most specialists provided additional information or resources in the eConsult, only one suggested completion of a BPMH. Gathering a BPMH is a necessary first step toward deprescribing, and conducting a structured medication review could be a viable way to help minimize PIM use in older adults with frailty [2]. Deprescribing in older adults with frailty may lead to mental status improvements and a decrease in potential adverse drug reactions and geriatric depression scale scores [21]. Deprescribing interventions in which pharmacists provide recommendations to PCPs based on medication reviews have been shown to decrease pill burden in older adults in long-term care [22]. Incorporating pharmacists regularly in eConsult could help deprescribing be considered and recommended when appropriate.

Within eConsult services, a recent study showed 75% of patients had at least 1 potentially clinically significant discrepancy involving a PIM between a clinician supplied medication list and a pharmacist-led BPMH [18]. Medication histories were included in 83% of cases in this study and may not have been complete; this could have led to specialists missing opportunities to recommend deprescribing and emphasizes the importance of an accurate medication history as a part of eConsults.

## Limitations

This exploratory study is not without limitations. There were a small number of clinicians, all pharmacists, involved in developing and testing the classification system. Medication histories provided in the cases could not be verified for completeness, and therefore patients could have been taking different medications not captured during analysis. Some questions were removed from the final classification system due to the lack of signals in the small number of cases in this study.

There is potential to apply this classification system to more cases to test its validity and reliability, identify other signals, and inform learning opportunities for PCPs and eConsult triage pathways. Service enhancements may include better leveraging geriatricians and pharmacists and offering comprehensive medication reviews to identify PIMs and support deprescribing [2]. The feasibility of the classification system could be further tested by including other clinicians and researchers to apply and validate the classification system. The classification system could also be refined to further explore medication-specific information like adverse events and specific recommendations such as lab tests, dose changes, and patient education, which were not captured due to the small sample of cases [16].

## Conclusion

This study proposed and tested the feasibility of a classification system that can be used to describe medication-related eConsults for older adults with frailty. Applying this classification system to a larger number of eConsult cases could help identify PCP learning opportunities and improve the eConsult service model.

### Electronic supplementary material

Below is the link to the electronic supplementary material.

**Supplementary Material 1: Table 1.** The Final Classification System Created to Classify Medication-Related eConsults and **Table 2.** Questions Removed From the Final Classification System If the editor’s preference is to list them together, the caption could be "Final Classification System Created to Classify Medication-Related eConsults and Removed Question

## Data Availability

The data used in this study cannot be publicly shared due to confidential patient information in the eConsult cases and analysis. Data without patient identifiable information will be shared by the corresponding author upon request.

## Abbreviations

AGS: American Geriatrics Society
ATC: Anatomical Therapeutic Chemical
BPMH: Best possible medication history
eConsult: Electronic consultation
PIM: Potentially inappropriate medication
PCP: Primary care provider
SD: Standard deviation
