# Supplementary material for: Classification system for primary care provider eConsults about medications for older adults with frailty
Source: BMC Prim Care. 2024 Apr 2;25:104. doi: 10.1186/s12875-024-02340-5 (PMC10985926; doi:10.1186/s12875-024-02340-5)
Supplement: Supplementary file 1 — Supplementary Material 1: Table 1. The Final Classification System Created to Classify Medication-Related eConsults and Table 2. Questions Removed From the Final Classification System If the editor’s preference is to list them together, the caption could be "Final Classification System Created to Classify Medication-Related eConsults and Removed Question [file 12875_2024_2340_MOESM1_ESM.docx]

**SUPPLEMENTARY MATERIAL**

**Title**: Classification System for Primary Care Provider eConsults About Medications for Older Adults with Frailty

**DOI**: 10.1186/s12875-024-02340-5

**Authors**: T Schneider, B Farrell, S Karunananthan, A Afkham, E Keely, C Liddy, LM McCarthy

Supplemental Table 1: The Final Classification System Created to Classify Medication-Related eConsults

| Number | Question | Explanation | Section |
| --- | --- | --- | --- |
| 1 | Case Identifier | Case number. | Case descriptives |
| 2 | Patient age | Age in years. | Case descriptives |
| 3 | Patient sex | Code as male/female/unknown. | Case descriptives |
| 4 | PCP requesting eConsult | Code as physician or nurse practitioner. | Case descriptives |
| 5 | Specialist consulted | e.g., endocrinology, cardiology. | Case descriptives |
| 6 | Does the PCP inquire about a medication or class? | Code as yes/no.   - A medication would be any pharmacologically active substance including over the counter medications, natural health products. | Intent and type of question |
| 7 | Does the PCP ask for general advice on additional treatment options? | Code as yes/no.  Code yes if:   - PCP is asking for general recommendations about other treatments. | Intent and type of question |
| 8 | Number of pharmacological options inquired about? | Include specific medications/classes.   - Add 1 per specific medication or class mentioned. - If a class and medication(s) within that class are mentioned, count as 1 (e.g., opioids like hydromorphone and morphine = 1). - Include pharmacological agents as found on the ATC classification, e.g., enema is found on ATC so it is included, transanal irrigation is not on ATC and not included. | Intent and type of question |
| 9 | Did the PCP propose a plan and asked for agreement? | Code as yes/no. | Intent and type of question |
| 10 | Did the PCP propose a plan and ask for additional options? | Code as yes/no. | Intent and type of question |
| 11 | Did the PCP not propose a plan and just ask for advice? | Code as yes/no. | Intent and type of question |
| 12 | Did the specialist recommend a medication/class? | Code as yes/yes (if you want)/no.   - If the specialist recommended deprescribing, code as yes/yes (if you want) depending on the enthusiasm/strength of the recommendation. | Medication recommendations and additional information in response |
| 13 | Number of pharmacological options recommended | Include specific medications/classes.   - Add 1 per specific medication or class mentioned. - If a class and medication(s) within that class are mentioned, count as 1 (e.g., opioids like hydromorphone and morphine = 1). - Include pharmacological agents as found on the ATC classification, e.g., Enema is found on ATC so it is included, transanal irrigation is not on ATC and not included. - Include recommendations from follow-up consultations. - Do not include recommendations for deprescribing here. | Medication recommendations and additional information in response |
| 14 | Did the specialist agree with PCP about the medication/class inquired? | Code as yes/yes in addition to inquired medication/no/PCP did not inquire about a specific medication and list the recommendation.  Code “yes” if:   - PCP asks if a medication should be stopped and the specialist says yes. - PCP asks to start a specific medication and the specialist recommends that medication. - PCP asks about a class (e.g., proton pump inhibitor) and a specialist recommends a medication in that class (e.g., omeprazole). - PCP asks about a specific medication (e.g., omeprazole) and the specialist recommends that class (e.g., proton pump inhibitor). - If PCP inquires about 2 options and asks which one, and the specialist answers yes to one or both.   Code “no” if:   - If PCP asks about a specific medication (e.g., omeprazole) and the specialist recommends a different medication in that class (e.g., pantoprazole), answer no.     Code yes in addition to inquired medication if:   - PCP asks about a medication or class (e.g., proton pump inhibitor), and the specialist recommends that medication or class or something else (e.g., proton pump inhibitor or histamine type-2 receptor antagonists). | Medication recommendations and additional information in response |
| 15 | Was there a different medication/class recommended than the PCP inquired about? | Code as yes/yes in addition to inquired medication/no/PCP did not inquire about a specific medication/did not recommend a medication and list the recommendation.   - See coding instructions in question 10. | Medication recommendations and additional information in response |
| 16 | Was non-pharmacological treatment explicitly inquired about? | Code as yes/no and list the treatment inquired.   - Include testing, monitoring, referrals, self-care or supportive therapy, etc. - Do not include pharmacologically active substances (e.g., over-the-counter products, natural health products). | Intent and type of question |
| 17 | Was non-pharmacological treatment recommended? | Code as yes/no and list the treatment recommended.   - Include testing, monitoring, referrals, self-care or supportive therapy, etc. - Do not include pharmacologically active substances (e.g., over-the-counter products, natural health products). - Do not include recommendations to avoid medications. | Medication recommendations and additional information in response |
| 18 | Did the specialist recommend starting a new medication/class? | Code as yes strong recommendation/yes recommended as an option/no and list the recommendation. | Medication recommendations and additional information in response |
| 19 | Did the specialist recommend stopping a medication/class? | Code as yes strong recommendation/yes recommended as an option/no and list the recommendation. | Medication recommendations and additional information in response |
| 20 | Did the specialist recommend avoiding any medications? | Code as yes/no and list the recommendation.   - Include recommendations to deprescribe. | Medication recommendations and additional information in response |
| 21 | Did the specialist recommend doing something if disease recurrence or progression occurs? | Code as yes/no and list the recommendation. | Medication recommendations and additional information in response |
| 22 | Did the specialist mention current drugs taking that might contribute to signs/symptoms? | Code as yes/no and list the drug(s). | Medication recommendations and additional information in response |
| 23 | Was there a follow up consultation? | Code as yes/no.   - Include medications inquired and recommended in the follow-up consults in all questions.   Code as yes if:   - PCP asked another question. - Provided more information to the specialist. | Intent and type of question |
| 24 | What was the nature of the follow up consultation? | Briefly explain.   - e.g., clarification, drug coverage, further information provided to the specialist, additional questions asked. | Intent and type of question |
| 25 | Total number of recommendations | Code as a number (recommendations for pharmacological + non-pharmacological + avoid).   - A recommendation for non-pharmacological management (e.g., diet, exercise, fall prevention) would be 3 recommendations, monitoring would be 1 recommendation. - Include recommendations to avoid meds (e.g., avoid anticholinergics and opioids would be 2 recommendations, stop drug x and start drug y would be 1 recommendation as this is a recommendation to switch medications). - If a class and medications within that class are mentioned, count it as 1 (e.g., opioids like hydromorphone and morphine = 1). | Medication recommendations and additional information in response |
| 26 | Number of medications the patient is taking | Code as a number or N/A if no medication list is provided.   - Include the number of medications provided in the consult or available in any attached notes, even if it may not be complete to have the most information possible for coding (use the most recent comprehensive medication list, avoiding duplicate prescriptions (brand and generic for the same medication) if using pharmacy dispensing records). | Case descriptive |
| 27 | ATC for drug inquired about | List codes for all drugs inquired about separated by commas.   - e.g., G04CA01. - If inquiring about a class and giving an example, use ATC code of the class (e.g., opioid like morphine, use ATC code for opioid). | Medication classification |
| 28 | Classification of 1st level | e.g., G. | Medication classification |
| 29 | Classification of 2nd level | e.g., G04. | Medication classification |
| 30 | Classification of 3rd level | e.g., G04C. | Medication classification |
| 31 | Classification of 4th level | e.g., G04CA. | Medication classification |
| 32 | ATC recommended by specialist | List codes for all drugs recommended separated by commas.   - e.g., G04CA01. - If the specialist recommends a drug but also recommends a 2nd line drug if progression/recurrence then include both drugs. - If recommending a class and giving an example, use ATC code of the class (e.g., opioid like morphine, use ATC code for opioid). - If a specialist recommends to deprescribe a medication, it should not be listed here. | Medication classification |
| 33 | Classification of 1st level | e.g., G. | Medication classification |
| 34 | Classification of 2nd level | e.g., G04. | Medication classification |
| 35 | Classification of 3rd level | e.g., G04C. | Medication classification |
| 36 | Classification of 4th level | e.g., G04CA. | Medication classification |
| 37 | If medication list provided, how many drugs on AGS Beers Criteria® (Table 2) is the patient taking? | Code as name of drug(s) or N/A.   - Include the drug if it appears on the medication list and Table 2 of AGS Beers Criteria®; do not make a decision on if it is clinically appropriate. | PIMs |
| 38 | Is a drug on AGS Beers Criteria® inquired about? | Code as name of drug(s)/no/ N/A.   - Select N/A if a drug was not inquired about in the consultation. - Include the drug if it appears on the medication list and Table 2 of AGS Beers Criteria®; do not make a decision on if it is clinically appropriate.   Include the drug if it appears on the AGS Beers Criteria® and the PCP is asking for tapering instructions. | PIMs |
| 39 | Is a drug on AGS Beers Criteria® recommended? | Code as name of drug(s)/no/ N/A.   - Select N/A if a specific medication/class was not recommended in the consultation. - Include the drug if it appears on the medication list and Table 2 of AGS Beers Criteria®; do not make a decision on if it is clinically appropriate. - Do not include if the specialist recommends deprescribing of the drug. | PIMs |
| 40 | STOPPFall medications in med list? | Code as name of drug(s) and the STOPPFall class/no/ N/A.   - Find the classes in Figure 2 of STOPPFall. - Use AGS Beers Criteria® for the list of anticholinergic drugs for the anticholinergic category of STOPPFall as STOPPFall does not provide a list of anticholinergics. - If a medication can go under 2 categories, only code for the highest category to avoid double counting. - Include the drug if it appears on the medication list and Figure 2 of STOPPFall; do not make a decision on if it is clinically appropriate. | PIMs |
| 41 | Is a medication on STOPPFall inquired about? | Code as yes and the medication/no/ N/A if the PCP did not inquire about a medication. | PIMs |
| 42 | Is a medication on STOPPFall recommended? | Code as yes and the medication/no/ N/A if the specialist did not recommend a medication. |  |
| 43 | ACB Scale score of medications patient is taking | Code as a number or N/A if there is not a medication list provided.   - Use acbcalc.com for the ACB scale score. | PIMs |
| 44 | ACB Scale score of medication inquired | Code as a number or N/A.   - If a specific medication is not inquired about (i.e., a medication class), then code as N/A. - If a class is inquired about but a specific medication is mentioned, search for that medication (e.g., opioids like morphine - search morphine). | PIMs |
| 45 | ACB Scale score of medication recommended | Code as a number or N/A.   - If a specific medication is not recommended (i.e., a medication class), then code as N/A. - If a class is recommended but a specific medication is mentioned, search for that medication (e.g., opioids like morphine - search morphine). | PIMs |
| 46 | ThinkCascades: does this consult involve a patient taking medications that could be a clinically important prescribing cascade? | Code as yes/no.   - Include the cascades if it appears on the medication list and ThinkCascades; do not make a decision on if it is clinically appropriate. | PIMs |
| 47 | Which cascade is involved? | Code as yes and list the cascade or N/A. | PIMs |
| 48 | How was the eConsult answered? | Select best option: reassured correct therapy (agree with inquiry), agreed with one of the options presented, recommended additional treatment, recommended different drugs/treatment than inquired, recommended drug they are on already but different dosing, recommended discontinuing drug/deprescribing, monitoring.   - If PCP proposes 2 options and the specialist only agrees with one, select agreed with one of the options presented. - If there is not an inquiry about a specific drug and the specialist recommends something, select “recommended additional treatment”. | Medication recommendations and additional information in response |
| 49 | Form state (Outcome of the consult) | Code as completed (PCP declined more info), completed (PCP accepted recommendation), etc.   - Found at the end of eConsult. | Case descriptives |
| 50 | Was additional advice provided by the specialist or did they just answer the question? | Code as additional advice provided and briefly explain/ specialist just answered the question. | Medication recommendations and additional information in response |
| 51 | Were guidelines/articles/tools referenced or provided? | Code as yes and list the reference/ no. | Medication recommendations and additional information in response |
| 52 | Any comments a pharmacist/reviewer would provide? | Code as yes and briefly explain/ no. | Case descriptives |
| 53 | Miscellaneous Notes | Free form/ N/A. | Case descriptives |

ACB - Anticholinergic Cognitive Burden

AGS - American Geriatrics Society

ATC - Anatomical Therapeutic Chemical

PCP - primary care provider

N/A - not applicable

Supplemental Table 2: Questions Removed From the Final Classification System

| Question | Explanation | Section | Reason for Removal |
| --- | --- | --- | --- |
| Which drug therapy problem category does the question fall under? | Additional drug therapy, unnecessary drug therapy being used, ineffective drug therapy, dosage too low, adverse drug reaction, dosage too high, adherence to therapy.   - Apply hierarchy of Indication, Efficacy, Safety, Adherence. | Intent and type of question | Cases with multiple questions became hard to code. Some questions covered multiple drug therapy problems, and if going off of the hierarchy then most cases would be coded as indication. |
| Classification of question type? | List all that apply:   - Diagnosis. - Therapeutics. - Disease prevention/ health education. - Prognosis. | Intent and type of question | This measure could be somewhat subjective, and by listing all it could have included some types that were not fully relevant to the question. Additionally, picking the most appropriate type instead of listing all was difficult. |
| If med list, how many drugs on AGS Beers Criteria® for Potentially Inappropriate Medications: Drugs To Be Used With Caution in Older Adults is the patient taking? | Code as name of drug(s) or N/A.   - Include the drug if it appears on the medication list and the Drugs to be Used With Caution list from AGS Beers Criteria®; do not make a decision on if it is clinically appropriate. | PIMs | This could be confusing to readers who are not aware there are multiple tables included in the AGS Beers Criteria®. |
| Is this consult asking about optimizing current therapy? | Code as yes/no. | Intent and type of question | It was difficult to get a clear definition for optimizing therapy, where most of these cases could be classified as optimizing therapy. |
| What is the content of the question? | Code as:   - Reassurance (asking if already on best treatment or additional suggestions without offering a proposed next step). - Problem caused by a drug. - Optimization of therapy (right drug vs different drug (e.g., PCP offers suggestions for the next step), dosing)). | Intent and type of question | It was difficult to standardize between cases as what constituted reassurance compared to optimization of therapy as some cases could be classified under many of these categories. |
| Could the eConsult have gone elsewhere? | Code as yes and briefly explain/ no.   - e.g., to a pharmacist/ other specialty. | Case descriptives | This was quite subjective to the coder and difficult to standardize across cases. |

AGS - American Geriatrics Society
